# Comprehensive Surgical Management of a Carotid Body Tumour: A Case-Based Approach

**DOI:** 10.12669/pjms.42.2.13331

**Published:** 2026-02

**Authors:** Mohamed Mostafa Elshamaa, Alaa Abdelhamid, Shaul Hameed Kolarkodi, Khalid Alotaibi, Samir Ali Elborolosy

**Affiliations:** 1 Mohamed Mostafa Elshamaa Associate Professor, Oral and Maxillofacial Surgery Department, Faculty of Dentistry, Beni-Suef University, Egypt; 2 Alaa Abdelhamid Professor, Department of Periodontology and Implant Dentistry, College of Dentistry, Qassim University, Saudi Arabia; 3 Shaul Hameed Kolarkodi Assistant Professor, Department of Oral and Maxillofacial Diagnostic Sciences, College of Dentistry, Qassim University, Saudi Arabia; 4 Khalid Alotaibi Assistant Professor, Department of Oral and Maxillofacial Surgery, College of Dentistry, Qassim University, Saudi Arabia; 5 Samir Ali Elborolosy Associate Professor, Oral and Maxillofacial Surgery Department, Faculty of Dentistry, Beni-Suef University, Egypt. College of Dentistry, Qassim University, Saudi Arabia

**Keywords:** Carotid Body Tumour, Head and neck tumor, Imaging, Paraganglioma, Surgical Management, Shamblin Classification, Vascular Neoplasm

## Abstract

A carotid body tumor (CBT) is a head and neck tumor, being the most frequent form of head and neck paraganglioma, which has its origin in specialized cells at the carotid bifurcation. They are benign, slow growing neuroendocrine neoplasia that are difficult to manage because they are located vascularly and may cause complications that make it difficult to be successfully managed without a multidisciplinary approach to the treatment. CBTs are uncommon, highly vascular tumors which develop out of the neural crest-derived paraganglia in the carotid bifurcation. They are generally harmless, but they are mostly difficult to manage due to their positioning in crucial neurovascular areas. Diagnosis should be made early as they may exhibit malignant transformation and local aggressive invasion. We present a case of a 51 years old woman who has been successfully excised of a mass in the carotid body using the multidisciplinary approach to ensure the best patient survival. The current case report is consistent with the literature results, which state that preoperative assessment, surgical planning, and a team-based way of work are crucial to the management.

## INTRODUCTION

Paraganglioma tumours which are known as carotid body tumours occur in the chemoreceptor cells of the carotid body. They may be either parasympathetic or sympathetic, majority of which are non-functional. These tumours rarely secrete catecholamines and mimic pheochromocytoma with such symptoms as tachycardia and hypertension; therefore, modern methods of diagnosis have replaced carotid arteriography with non-invasive imaging methods such as Magnetic Resonance Angiography (MRA) or Duplex Ultrasound scanning.^1^ CBTs are surgical dilemmas because of their hyper vascularity and close anatomical proximity to external and internal carotid arteries. Personalized surgery, in most cases with the help of a multidisciplinary team, is necessary to reduce complications and guarantee that the tumour is removed successfully. More recent research indicates that the size of tumours, vascular encasement, and pre-operative embolization have a great influence on the outcomes of surgery.^2^

## CASE PRESENTATION

**Fig.1:**
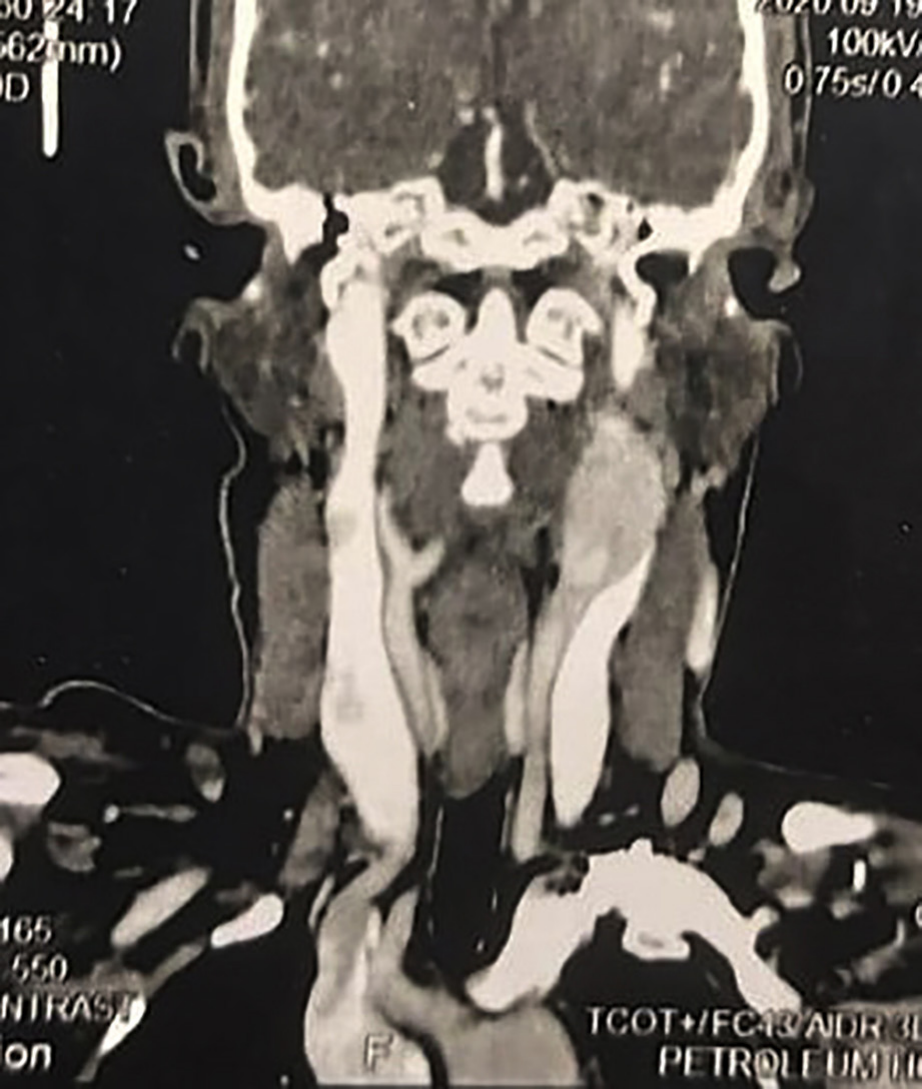
CT Angiography of Carotid Body Tumour Showing Vascular Involvement.

**Fig.2:**
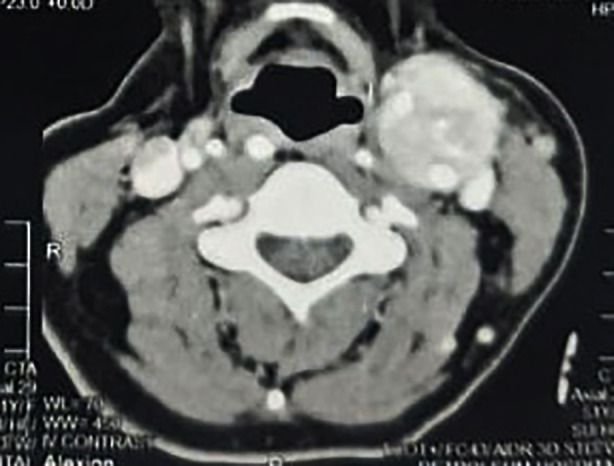
Axial CT Angiography Showing Carotid Body Tumour with Vascular Splaying.

A 51-year-old female, a housewife from Alexandria, presented with a progressive left-sided neck swelling. Clinical examination revealed a well-circumscribed, mobile swelling in the submandibular region. The swelling exhibited horizontal mobility but was fixed in the vertical plane (positive Fontain sign). The patient had a history of left submandibular sialoadenectomy for presumed sialolithiasis a year earlier. Multi-detector contrast-enhanced CT imaging of the neck revealed a hypervascular soft tissue mass (4×3.5 cm) at the left carotid bifurcation, splaying the internal and external carotid arteries and encasing their proximal segments (Fig.1,2). The lesion exhibited intense post-contrast enhancement, characteristic of a carotid body tumour. A balloon occlusion test was positive, confirming the feasibility of vascular intervention if required.

**Fig.3:**
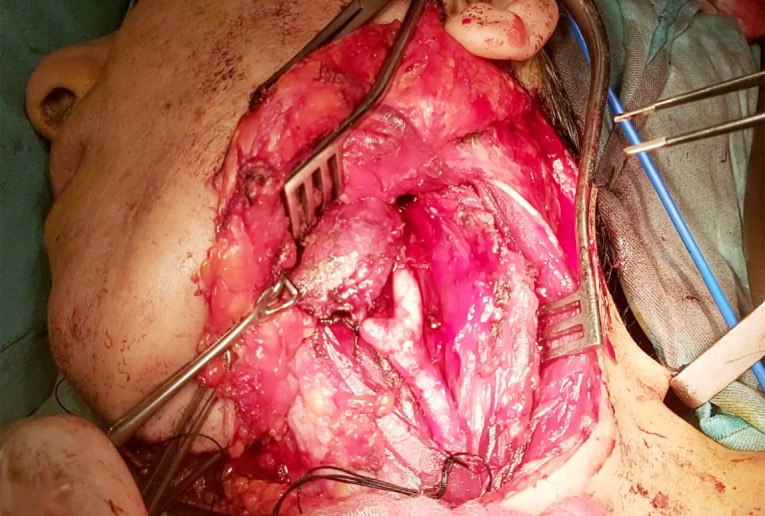
Intraoperative Image of Carotid Body Tumour Excision with Vascular and Nerve Exposure.

**Fig.4:**
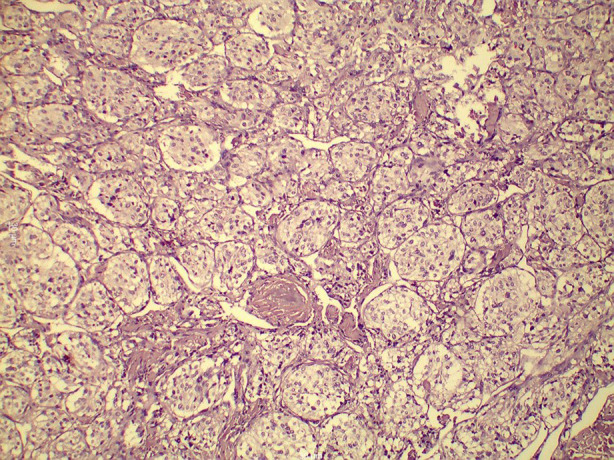
Histopathological Image of Carotid Body Tumour Demonstrating Characteristic Nesting Architecture (Zellballen) Within a Prominent Vascular Network (H&E, ×200 Magnification).

**Fig.5:**
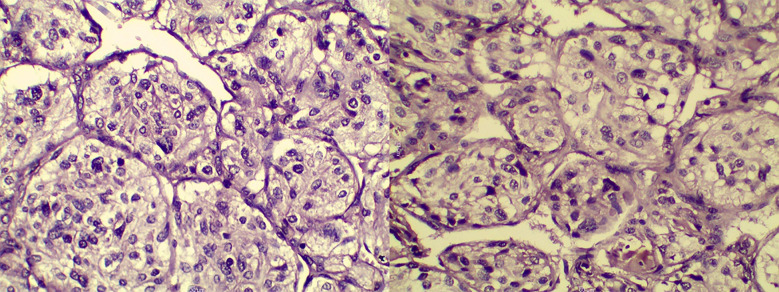
Higher Magnification of the Previous Photomicrograph Revealing Oval Cells and Giant Multinucleated Cells With Abundant Granular Eosinophilic or Basophilic Cytoplasm.

A surgical approach was meticulously planned. Initially, an access mandibulotomy was considered for exposure; however, adequate tumour excision was achieved via a mid-neck incision without the need for mandibulotomy or lip splitting. The external carotid artery was ligated, but no vascular reconstruction was required. The tumour was classified as Shamblin II (Fig.3). Histopathological examination confirmed the diagnosis of a glomangioma (CBT), showing characteristic nesting (Zellballen) architecture within a prominent vascular network (Fig.4). Higher magnification revealed oval cells with multinucleated giant cells and eosinophilic or basophilic cytoplasm (Fig.5). Postoperative recovery was uneventful, and follow-up imaging showed no recurrence. The patient remains asymptomatic, emphasizing the success of the surgical strategy.

## DISCUSSION

The most common head and neck paragangliomas are carotid body tumours (CBTs), which are rare but highly vascular neuroendocrine neoplasms that develop at the carotid bifurcation. They are generally non-malignant but have a predisposition rate of 1-2 in 100,000, there is a mild female predisposition, and they may be intermittent or connected with family SDH gene mutations.^3^ Transverse cervical incision is the desirable method of excising tumors of the carotid body as it has the best exposure, better visualization of vital structures and greater hemostatic control than the traditional vertical SCM-border incision, although it leaves a more visible scar. Following the elevation of subplatysmal flaps, sparing of the great auricular nerve, and the external jugular vein is ligated in case the need arises, the investing layer of the deep cervical fascia is incised and the SCM is pulled backwards to expose the carotid sheath. This is followed by blunt dissection to discover the common carotid artery and track it up to bifurcation, taking care to ligate tributaries of the veins. The tumor is carefully excised off the arterial wall in a controlled exposure and hemostasis is ensured before layered closure. The possible complications are carotid blowout- an intraoperative emergency, which necessitates instant clamping and vascular repair, injuries to the external carotid artery and injuries to the internal or common arteries that demand immediate reconstruction, most commonly with the help of stents or vein grafts in patients who fail preoperative balloon occlusion testing. When there is insufficient exposure, e.g. short-necked patient or tumor moved under the mandible, a mandibulotomy with plating can be necessary so that the tumor is removed safely and completely.^4^

Various studies highlight the need to have close preoperative planning and early detection of surgical risks to minimize surgical risks in carotid body tumours (CBTs). According to a systematic review by Alanezi et al. (2024), the majority of the pediatric cases were diagnosed as painless progressive neck swellings, which often delayed the diagnosis, whereas Shankar et al. (2017) illustrated the complexity of large Shamblin III tumours that could only be treated with a combination of sternotomy and mandibulotomy- which was not the case with the current case as it was fully treated using a mid-neck incision without any reconstruction.^5^,^6^ Preoperative embolization is recommended, but the usefulness of this practice remains controversial: the study by Lebbe et al. (2020) showed that it was helpful in the case of Shamblin II–III tumours, and the embolization was not applied in our case, which did not result in excessive blood loss.^7^ Histopathological examination, such as the typical Zellballan pattern and positive immunoreactivation of such markers as S-100, synaptophysin and chromogranin were consistent with previous literature.^2^ The literature of postoperative complications, which mainly did not persist, i.e., temporary cranial nerve deficits like dysphagia or vagal dysfunction, did not occur in our case, which demonstrates the importance of a nerve-sparing technique. Prognosis is usually positive, but malignant transformation can happen in up to six percent of cases, which is why it is necessary to follow all patients with long-term imaging, which is especially relevant in patients with a familial or SDH-associated risk factor.^2^,^8^

## CONCLUSION

Carotid body tumours are not very common but once diagnosed early and with a detailed surgical plan, the tumours can be managed successfully. The current case highlights the importance of multidisciplinary collaboration, a thorough preoperative assessment, and surgical accuracy in delivering superior results with the lowest number of complications. The case, as supplemented with the findings of recent studies, establishes the importance of imaging, embolization, and careful surgical planning in the treatment of CBTs.

### Authors` Contribution:

**MME and SAE** were involved in patient care, data collection, and manuscript drafting.

**AA** contributed to the conception and design of the study.

**KA** assisted in the critical revision of the manuscript.

**SHK** played a key role in the overall coordination and drafting of the manuscript.

All authors **(AA, MME, SAE, KA, SHK)** have critically reviewed and approved the final version of the manuscript. All authors have critically reviewed and approved the final draft and are responsible for the content and similarity index of the manuscript.
